# Epithelioid angiomyolipoma of the kidney in an adult male with tuberous sclerosis

**DOI:** 10.1016/j.eucr.2022.102204

**Published:** 2022-09-05

**Authors:** Nada Shaker, Douglas Wu, Anil Parwani

**Affiliations:** a Department of Pathology, The Ohio State University Wexner Medical Center, E322 Doan Hall, 410 W. 10th Ave, Columbus, OH, 43210, USA; b The Ohio State University Wexner Medical Center, E322 Doan Hall, 410 W. 10th Ave, Columbus, OH, 43210, USA

## Abstract

Epithelioid angiomyolipoma is a rare variant of angiomyolipoma and a part of the microphthalmia transcription factor (MiTF) tumors. We report a case of epithelioid angiomyolipoma of the kidney that occurred in a 40-year-old male. The tumor was composed of sheets of uniform epithelioid cells with clear to granular eosinophilic cytoplasm and hyperchromatic nuclei. The tumor extended into the vascular margin and perinephric fat with identified angiolymphatic invasion. Immunohistochemistry showed diffuse staining for HMB45, Melan A, and focal staining for SMA. No expression for EMA, AE1/AE3, CK7, S100, or Desmin was noted. The patient underwent a left nephrectomy with no recurrence.

## 1 Introduction

Epithelioid angiomyolipoma (EAML) is a rare variant of angiomyolipoma that is a part of a family of neoplasms derived from perivascular epithelioid cells and a part of a microphthalmia-associated transcription factor (MiTF) family of tumors. It is defined by the presence of round to polygonal cells with abundant pink to eosinophilic/clear cytoplasm and varying degrees of nuclear atypia.^1^^,^^2^ It is frequently associated with tuberous sclerosis (TSC).^3^ Mean age of presentation is middle age 50 years with a male-to-female ratio of 9:11.^1^ The most common site is the kidneys, but it can also affect the liver, urinary bladder, adrenal gland, and nasal cavity or skin.^1^ It is usually asymptomatic and discovered incidentally on imaging and often indistinguishable from renal cell carcinoma on radiology. This neoplasm is generally characteristically positive for melanoma markers (cathepsin K, HMB45, Melan A, MiTF) and negative for PAX8, cytokeratins, S100, and SOX10. The prognosis is potentially malignant and can recur and metastasize to distal organs with a rate ranging from 5% to 45%.^1^ Surgical resection is the optimal treatment choice and mTORC1 targeted therapy inhibitors, such as Everolimus and Sirolimus have been used in some cases.

### 1.1 Case presentation

**Fig. 1:**
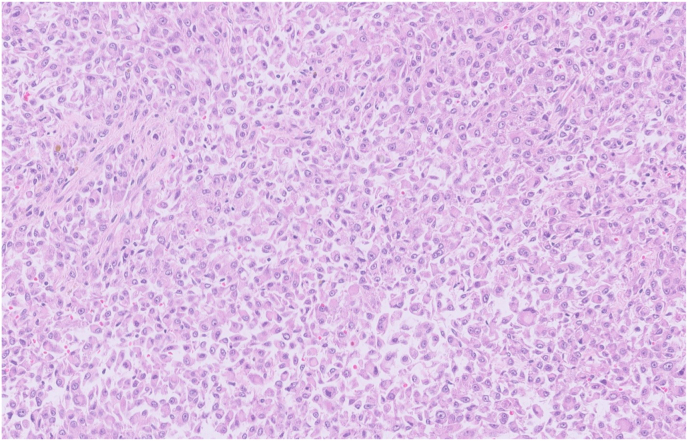
Hematoxylin and Eosin staining of renal epithelioid angiomyolipoma of the left kidney, high power field magnification ×40 demonstrates tumor cells with abundant pink granular cytoplasm and nuclear pleomorphism. (For interpretation of the references to colour in this figure legend, the reader is referred to the Web version of this article.)

**Fig. 2:**
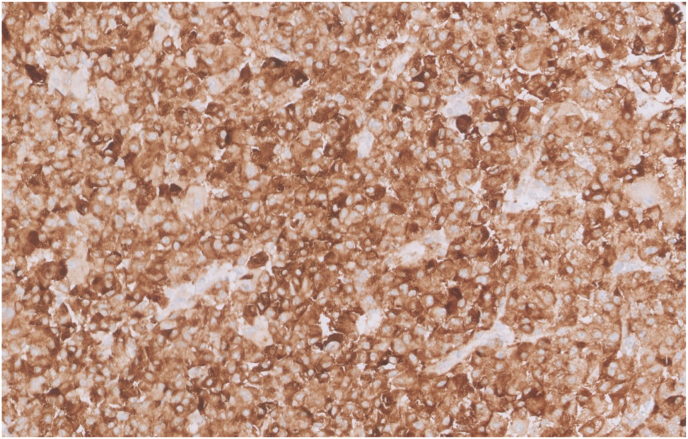
Immunohistochemical staining of Melan A shows diffuse cytoplasmic expression.

**Fig. 3:**
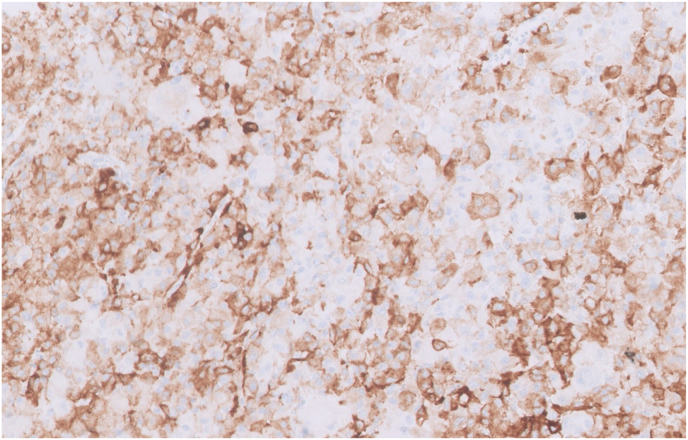
Immunohistochemical staining of HMB45 shows cytoplasmic expression in tumor cells.

A 40-year-old male with a past medical history of tuberous sclerosis presents with 5 months of painless gross hematuria and mild left mid quadrant abdominal pain. Abdominal MRI showed a large left renal mass with a central stellate scar, measuring 16 × 14 × 11 cm. The patient underwent a left nephrectomy, and the tissue was sent for histopathological examination. Under microscopy, tumor cells were relatively uniform epithelioid cells with clear to granular eosinophilic cytoplasm. Fig. 1. Epithelioid cells were arranged in densely packed diffuse sheets. The tumor extended into the vascular margin of resection and the perinephric fat. The angiolymphatic invasion was present. Immunohistochemistry showed diffuse strong staining for HMB45, Melan A, and focal moderate staining for SMA. Fig. 2, Fig. 3. The tumor cells showed negative staining for EMA, AE1/AE3, CK7, S100, and Desmin. Necrosis was present in approximately 30% of tumor cells.

## 2 Discussion

Epithelioid angiomyolipomas are a rare variant of angiomyolipoma (<5% of all angiomyolipoma cases) that demonstrate predominant epithelioid histology (>80%).^1^^,^^4^ Unlike classical angiomyolipomas, EAMLs are potentially malignant with the capability of recurrence and metastasis.^1^^,^^5^ They occur in a wide age range, 17–80 (mean 50 years), and are more common in females with an F: M ratio reported as 11:9.^1^ They usually present as asymptomatic masses discovered incidentally on imaging, although they can present with abdominal pain, hematuria, or palpable masses. Grossly, epithelioid angiomyolipomas are large (range 1–25, mean 8.7cm), predominantly solid pink-tan masses frequently containing areas of hemorrhage and necrosis.^1^ Borders can range from well-circumscribed to infiltrative, with possible extrarenal extension and involvement of renal veins. Malignant behavior can be predicted to some extent using a model of 4 atypical features including ≥70% atypical epithelioid cells, ≥ 2 mitotic figures per 10 high power field, atypical mitotic figures, and necrosis. The presence of 3 or 4 of these features is highly predictive of malignant behavior.^5^ Histologically, they are at least 80% epithelioid.^1^^,^^4^ Two histological patterns have been described, with some tumors demonstrating a variable combination of the two carcinoma-like growth patterns: cells are arranged as cohesive nests, broad alveoli, and compartmentalized sheets separated by thin vascular-rich septae. Cells typically appear as large and polygonal with dense, deeply eosinophilic cytoplasm.^4^ Nuclei appear atypical with nuclear pleomorphism, hyperchromatism, and prominent nucleoli. The combination of prominent nucleoli with intranuclear inclusions, in large discohesive cells with eosinophilic cytoplasm, imparts a ganglion cell-like appearance. Epithelioid and plump spindled cells in diffuse growth: epithelioid and plump spindled cells are arranged in densely packed diffuse sheets without compartmentalization by vascular separation. Cells are relatively uniform epithelioid cells with clear-to-granular, feathery eosinophilic cytoplasm. Nuclei are more homogeneous and lack atypia.

Immunohistochemistry staining demonstrates tumor cells that are positive for cathepsin K, HMB45, Melan A, microphthalmia transcription factor (MITF), tyrosinase, CD68, and TFE3.

Tumors are variably positive for SMA. Tumors are negative for PAX8, cytokeratins (AE1/AE3, CAM5.2, EMA), S100, and SOX10. The molecular profile associated with these tumors reveals a loss of heterozygosity of chromosome 16p (TSC2) that has been reported in some cases.^2^

Similar to classic angiomyolipoma may or may not be associated with tuberous sclerosis complex (TSC) which is caused by inactivating losses of TSC1 (9q34) or TSC2 (16p13.3). A previous meta-analysis of 69 well-documented cases of EAML showed that 38% were malignant.^1^

## 3 Conclusion

Epithelioid angiomyolipomas are rare and potentially malignant with the tendency for recurrence and frequent metastasis. EAMLs may be misdiagnosed as malignant renal neoplasm on incidental radiology evaluation.
